# Measuring the quality of self-care of elderly patients with dementia in a developing country

**DOI:** 10.20935/mhealthwellb7300

**Published:** 2024-07-31

**Authors:** Dennis Rogers Buwembo, Joy Louise Gumikiriza-Onoria, Lwere Kamada, Mark Kaddu-Mukasa, Joseph Kagaayi, Juliet Kiguli, Martha Sajatovic, Noeline Nakasujja, Fredrick Makumbi

**Affiliations:** 1 Brain Health Training Program, School of Medicine, College of Health Sciences, Makerere University, Kampala, Uganda.; 2 Department of Psychiatry, School of Medicine, College of Health Sciences, Makerere University, Kampala, Uganda.; 3 Department of Microbiology, School of Biomedical Sciences, College of Health Sciences, Makerere University, Kampala, Uganda.; 4 Department of Internal Medicine, School of Medicine, College of Health Sciences, Makerere University, Kampala, Uganda.; 5 Department of Epidemiology and Biostatistics, School of Public Health, College of Health Sciences, Makerere University, Kampala, Uganda.; 6 Department of Community Health and Behavioral Sciences, School of Public Health, College of Health Sciences, Makerere University, Kampala, Uganda.; 7 Neurological and Behavioral Outcomes Center, School of Medicine, University Hospitals Cleveland Medical Center, Case Western Reserve University, Cleveland, OH, USA.

**Keywords:** dementia, quality, self-care, elderly-people, adaptation, validation

## Abstract

In developing countries like Uganda, people with dementia are cared for by non-medically trained family members with minimal support from the formal healthcare system. The quality of care in this setting is largely unknown but significantly affects the well-being of those with dementia. A tool designed to measure the quality of informal care for old frail adults with or without dementia was translated into Luganda. A committee of experts reviewed and finalized the translation, which was pilot-tested and then used to measure the quality of dementia self-care. We consecutively enrolled 105 caregivers of elderly people with dementia; the median age was 35 years (Interquartile Range 26–47 years), and 67% were females, taking care of a grandparent (44%) or a parent (34%). We used confirmatory factor analysis to assess for structural validity and computed correlation coefficients and Cronbach’s alpha to assess for discriminant validity and internal reliability, respectively. The three-factor model applied to the 20 items, adequately fit the data (Comparative Fit Index = 0.88, Tucker–Lewis Index = 0.87, Root Mean Square Error of Approximation = 0.08; 90% Confidence Interval (0.06–0.09), Standardized Root Mean Square Residual = 0.089). There was good discriminant validity, and correlation coefficients between dimensions/scales and the Dementia Knowledge Assessment Scale scores were low. There was good internal reliability with all items Cronbach’s alpha ranging from 0.69 to 0.89. Our findings demonstrated that this culturally adapted, shorter measurement tool is valid and reliable. The tool can be used by researchers, health workers, and agencies to assess the quality of self-care for elderly people with dementia in Uganda.

## 1. Introduction

Dementia is a major public and social health challenge worldwide. In 2019, dementia was the seventh leading cause of mortality, claiming about 1.6 million lives [1]. Dementia is among the leading causes of disability, dependence, and vulnerability to financial exploitation among elderly frail and cognitively impaired people [2]. Low- and middle-income countries bear the biggest burden of dementia, accounting for 60% of the 55 million people living with dementia. Dementia patients are projected to rise to 78 million by 2030 and 139 million by 2050 [3]. People with dementia experience a progressive decline in physical, memory, and cognitive functioning, leading to frequently changing complex care needs and increasing dependence [4]. Generally, management of people with dementia requires both medical and social care from formally trained health workers and informal care from untrained relatives, friends, and neighbors [5]. Informal care or self-care is when individuals, families, and communities prevent disease, cope with illness/disability, and promote and maintain health with or without the support of a trained health worker [6].

Globally, up to 84% of people with dementia receive care at home [5]. In sub-Saharan Africa, 96% of people with dementia rely on family, friends, and/or neighbors [5], with minimal formal support from the healthcare system [7]. In Uganda, over 80% of people with dementia live at home and are cared for by untrained family members, with less than 10% having access to paid caregivers [8]. The quality of care in this case is unknown, but poor self-care can lead to preventable hospital admissions for conditions such as un-intended self-harm, malnutrition, dehydration, and uncontrolled comorbidities. Yet, hospital admission puts elderly people with dementia at increased risk of harm and hastened death including mistreatment and exacerbation of dementia symptoms [9].

Quality assessments of dementia care typically focus on institutional settings such as nursing or care homes and trained health workers neglecting the unpaid family caregivers who provide most hands-on care [10]. There are no disease-specific tools to measure the quality of self-care of elderly people with dementia. Existing tools like the “Adequacy of Care (AoC)” assess the quantify of care provided for needs in instrumental and basic activities of daily living (ADL) but don’t consider—abuse and/or neglect [11]. The Exemplary Care Scale (ECS) extends the quality of self-care assessment to include assessing for sensitivity to the care recipient’s autonomy and wishes [12]. However, caregivers can still exhibit Potentially Harmful Behavior (PHB), using psychological acts, for example, yelling, screaming, name-calling, etc., and/or physical acts, for example, shaking, slapping, with-holding food on care recipients, most especially, when the desire is for the care recipient to follow caregivers’ or health workers’ instructions. Such acts, though not severe enough to require social services or legal intervention, are detrimental to the well-being of elderly people with dementia [13].

The tools discussed so far don’t encompass all aspects of the quality of self-care individually. Caregivers can offer varying degrees of support for ADLs and show sensitivity or insensitivity to care recipient’s preferences and sometimes exhibit PHB. A combined tool measuring support with ADL, sensitivity to care recipients’ needs for autonomy and PHB, was designed, psycho-metrically assessed, and used to measure the quality of self-care among frail elderly people with or without dementia [14]. Another assessment of the quality of self-care among people with dementia described six self-care giving styles, with three high quality-of-care styles (personalized, respectful, and compensatory) and three poor quality-of-care styles (punitive, controlling, and withdrawing) [15]. This study used the three-factor tool because it includes an assessment of support with ADL a critical issue among elderly people with dementia because of the significant contribution of dementia to dependency among people elderly people [16]. Relatedly, the styles of self-care described as poor quality of care by the six-factor tool are similar to what is measured by the PHB scale of the three-factor tool.

This study aimed to culturally adapt and validate the Luganda quality of self-care dementia assessment tool.

## 2. Materials and methods

### 2.1. Study participants and data collection

The study was conducted in the villages of Nansana municipality and Busukuma division of Wakiso district of Uganda. Data were collected between January and February 2024. A Village Health Team (VHT) member who knew caregivers of elderly people diagnosed with dementia contacted potential study participants to assess their willingness to participate in the study. Those who expressed willingness to participate in the study were consecutively enrolled if they were aged 18 years and above or an emancipated minor, self-identified as the primary caregiver, and could fully comprehend the Luganda language. Caregivers were excluded from participating in the study if they had any illness that could hinder their full participation in the study. Data collection involved interviewer-administered questionnaires. Interviewers were health workers with at least five years’ experience in conducting research interviews. They also went through training on the study questionnaire and study processes.

### 2.2. The original tool

The original tool, developed in English, is used to evaluate the quality of informal care for physically or cognitively impaired frail elderly in the United States. It consists of three-dimensional factors and 39 items: AoC (18 items), PHB (10 items), and ECS (11 items) [16]. The tool can be self-administered by people with mild to moderate dementia and their caregivers. For this study, trained interviewers administered the adapted Luganda version to caregivers.

### 2.3. Cultural adaptation of the quality of self-care measurement tool for people with dementia

We followed the guidelines for the process to cross-culturally adapt self-report measures as developed by Beaton et al. [17], to culturally adapt the questionnaire to Luganda. The processes are detailed below.

#### Forward translation to Luganda:

The tool was independently translated into Luganda by two native speakers proficient in English, one with a medical background and understood medical phrases and terms well.

#### Synthesis of the forward translated tool:

The translators and a recording observer (one of the researchers) synthesized the translations, noting and resolving discrepancies through consensus.

#### Back translation to English:

The synthesized Luganda tool was back-translated by two English proficient translators. Both have no medical background but have extensive experience in translating documents.

#### Expert committee review:

A committee of experts comprising two psychiatrists, a clinical Gero-psychologist, a medical doctor (generalist), an epidemiologist, one translator, a primary school teacher of English Language who is also a caregiver, and a researcher reviewed the translations for face and content validity. Items and responses in the tool were checked for relevance to the study population, purpose of application, and comprehensiveness. The committee ensured semantic, experiential, idiomatic, and conceptual equivalence was achieved. All expert committee members were proficient in English and all but one were proficient in Luganda. The committee prepared a report and a pre-final version of the Luganda questionnaire for pilot testing.

#### Pilot testing:

The pre-final tool was pilot-tested with 30 caregivers of people with dementia, who underwent cognitive interviews. The caregivers were asked to express their feelings about completing the questionnaire, indicate which questions were difficult to understand, and specify what was particularly challenging about those questions. They were also asked about the meanings of items and responses provided [18].

### 2.4. Measuring the quality of self-care for people with dementia

To assess AoC, caregivers were asked if in the past week the care recipient needed help in any basic and instrumental ADLs. If the care recipient needed help, caregivers were then asked how often they actually provided the help. Responses were captured on a four-point Likert scale (1—I never provided help, 2—sometimes I provide help, 3—I usually provide help, and 4—I always provide help). Responses were summed up, and an average based on the number of ADLs for which the care recipient needed help was computed to derive the total AoC score. Higher values reflected adequate or high quality of care, while the converse was true for inadequate or low quality of care. For PHB, caregivers were asked how often they had to use acts such as screaming, yelling that other caregivers use to compel care recipients to behave appropriately. Responses were recorded on a five-point Likert scale (0—never, 1—almost, 2—sometimes, 3—most of the time, and 4—all of the time). Responses were summed up to derive the total PHB score. Higher values reflect low quality of care and vice versa. For ECS, caregivers were asked to rate statements about their interactions with the care recipients. Responses were rated on a four-point scale (1—never, 2—sometimes, 3—often, and 4—always), and they were summed up and averages computed. Higher scores reflect high quality of care and the converse is true for low quality of care [14].

We also assessed caregivers’ knowledge about dementia using the Dementia Knowledge Assessment Scale (DKAS). A 25-item, four-factor instrument was used to measure dementia knowledge based on four domains: Causes and Characteristics, Communication and Behavior, Care considerations, and Risks and Health Promotion. The 25 questions about dementia are based on a literature review and an international Delphi study with dementia experts. Respondents answer the questions based on a modified five-point scale (1—false, 2—probably false, 3—probably true, 4—true, and 5—don’t know). Responses are summed up, the higher the score, the more knowledgeable one is about dementia. The tool has been shown to have a high internal consistency Cronbach’s alpha of 0.85 and high discriminant validity [19].

### 2.5. Validation of the instrument

Given that there is no gold standard for measuring the quality of self-care for elderly people with dementia, we assessed for content and construct validity. Content validity was assessed qualitatively by the committee of experts described above [20]. Construct validity was quantitively assessed using confirmatory factor analysis (CFA) through stata’s Structural Equation Modeling (SEM) to derive goodness of fit statistics, Comparative Fit Index (CFI), Tucker–Lewis Index (TLI), Root Mean Square Error of Approximation (RMSEA), and Standardized Root Mean Square Residual (SRMSR) [20–22]. To avoid missing values where a care recipient did not need help with an ADL, we adjusted the scoring for the AoC scale items, for which a care recipient did not need help to be scored as zero (0). Thus item scores for AoC ranged from 0 where no help with ADL was needed to 4 where help was needed and always provided. Discriminant validity was assessed using hypothesis testing. We set the hypothesis of a low correlation of ≤0.3 between the quality of care dimensions and scales and between the total quality of care score and the DKAS score. We computed Pearson’s correlation coefficients between dimension and factor scores and between the total quality of care score and the DKAS score. For internal reliability, we computed Cronbach’s alpha for items at the subscale and scale levels. All statistical analyses were performed using STATA version 15 (StataCorp LLC, College Station, TX, USA).

### 2.6. Ethical clearance

The study was cleared by the higher degrees committee of the School of Public Health, College of Health Sciences, and received ethical clearance from the Makerere School of Public Health, Research and Ethics Committee (MaKSPHREC), study registration number SPH-2022–354.

## 3. Results

### 3.1. Study participant characteristics

Table 1 summarizes the characteristics of study participants. In total, 105 caregivers of elderly people with dementia were consecutively enrolled. The median age was 35 years (IQR 26–47 years), with 67% being females. Most caregivers were looking after a grandparent (44%) followed by those looking after a parent (34%). Most had provided care for five or more years (32.4%) followed by those who had provided care for a period of one to two years (26.7%). Nearly all (93%) attended formal education, with 45% and 36% attending at least primary and secondary (O level) as the highest levels of education respectively. On the day of the interview, most reported to be in a fair general health condition (57.1%), but 44% indicated having a chronic illness. High blood pressure and chronic backache were the most commonly reported chronic illnesses each at 19.6%, followed by chronic intermittent headache at 15.2%. Of those with a chronic illness, 82.6% were actively seeking treatment; the majority (52.6%) sought care from private drug shops, while only 24% from government health facilities.

### 3.2. Content validity

The committee of experts unanimously affirmed that the questionnaire adequately reflected the construct to be measured. They agreed that the items are comprehensive and relevant to the study population in terms of age and disease characteristics. Moreover, there was consensus that the questionnaire could effectively distinguish between the quality of care provided by the caregivers at a point in time.

### 3.3. Cultural adaptation

During the synthesis of the forward-translated tools, the translators resolved discrepancies in items that were inconsistently translated or lacked direct equivalents in Luganda. For instance, the term “dementia” was initially translated as “Obulwadde bwo’ kuwutta obwongo” by one translator and as “obulwadde bwo’ kusimuuka obwongo” by another, respectively, meaning a disease characterized by loss of understanding and memory, or just memory loss. Additionally, there were variations in translating specific items like item 8 of the AoC scale, “During the past week, have you needed any help with transportation to places out of walking distance”, was translated as “needing help with transportation to places which are not near” (obuyambi n’okutuuka mu bifo ebitali byakumpi) by one translator, while the other translated the phrase as “needing help with transportation to places that are far and not within walking distance” (obuyambi bwona okumutambuzaako mu bifo ebyewala ebigere webitatuuka). This was harmonized to “needing help with transportation to far places where one is unable to reach by foot” (obuyambi, okumutwalako oba okumutusa mu bifo ebyewala omuntu watasobola kutambuza bigere).

### 3.4. Expert committee review

Cultural equivalence between source and target population encompasses semantic, idiomatic, experiential, and conceptual areas. The committee of experts made the following adjustments to the translated tool. Dementia had been translated as “Obulwadde bwo’kuwutta obwongo”. The committee agreed to replace “okuwutta” with “Okwelabiralabira” translating to forgetfulness, to avoid a derogatory connotation. Relatedly, the committee revised three items in the AoC scale and one item each in the PHB and ECS to improve clarity and cultural relevance. Changes included specifying “going outside the house”, modifying meal preparation roles based on gender, providing relevant examples for personal business tasks, replacing “nursing home” with “health facility” or “hospital”, and refining the description of a “bright and cheery place” to emphasize cleanliness and visual appeal. The committee of experts made changes to three items in the AoC scale, and one item each in the PHB and ECS. A detailed description of the changes is noted below.

#### Adequacy of Care (AoC)

- **Item 6**—“Getting outside” was modified to “going outside the house” in Luganda, “okufuluma ebweru w’enyumba”. The committee clarified that “outside” (“okufuluma”) could imply various actions, such as defecating or simply stepping outside the house, homestead, compound, or bedroom. Therefore, they specified it as “getting outside the house”.
- **Item 12**—“Preparing meals” had been translated to “cooking” (“Okufumba”). The committee noted that cooking is rarely done by men, so they modified the translation to “cooking” for women and “making plans” (“okutetenkanya”) for meals for men.
- **Item 15**—“Taking care of personal business such as insurance claims, taxes, etc.” The committee observed that it is uncommon for older adults in their setting to handle insurance claims or file taxes. They decided to provide examples such as getting a pension or accessing old age allowances.

#### Potentially Harmful Behavior (PHB)

- **Item 3**—“Threatened to take care recipient to a nursing home”. Since nursing homes are rare in Uganda.

#### Exemplary Care Scale (ECS)

- **Item 4**—“Makes sure the care recipient lives in a bright and cheery place”. Initially translated as “Omulwadde wabela walabikka bulungi, watemagana era nga wanyusa”, meaning looking good, sparkling, and appealing. The committee revised this to “walabika bulungi, wayonjo era nga wasanyusa”, meaning looking good, clean, and appealing to the eyes.

### 3.5. Pilot testing

Following pilot testing, no changes were made to the tool as all participants demonstrated a clear understanding of the questionnaire items. Responses were adequate, indicating comprehension and appropriateness of item meanings and responses.

### 3.6. Construct validity

Initially, CFA indicated a poor fit of the hypothesized three-factor model to the data. Subsequently, exploratory factor analysis (EFA) with orthogonal varimax rotation revealed a better fit for a reduced 20-item model. The CFA on this revised model demonstrated improved fit statistics (CFI = 0.884, TLI = 0.868, RMSEA = 0.077, SRMSR = 0.089), indicating satisfactory structural validity.

### 3.7. Hypothesis testing

Discriminant validity was supported by weak correlations between the three dimensions of the quality of dementia self-care tool (AoC with ECS = 0.06, ECS with PHB = 0.08, PHB with AoC = 0.2). Additionally, the correlation between the quality-of-care scores and the DKAS was found to be 0.086, supporting the hypothesized weak association (≤0.3) between the two constructs.

### 3.8. Internal reliability

Cronbach’s alpha values ranged from 0.687 to 0.89 for all items, indicating good internal consistency. Specifically, Cronbach’s alpha values were 0.89 for AoC, 0.87 for ECS, and 0.77 for PHB, respectively.

## 4. Discussion

We culturally adapted and validated the Luganda version of the Quality of Dementia Self-care Measurement tool. To our knowledge, this is the first attempt to tailor this tool to the Uganda and sub-Saharan context. Although Uganda has a diverse culture with different tribes, beliefs, value systems, and languages, Luganda, a Bantu language, is the most widely spoken language in the country. It is estimated that about 20 million people in Uganda and beyond speak Luganda due to factors critical for trade and business transactions [23].

By following stipulated guidelines for the cultural adaptation of self-report measures, we produced a shorter, valid, and reliable tool that can be used to measure the quality of dementia self-care. We are confident that the measurement tool developed can be well understood and used by people who comprehend Luganda. Forward and back-translations of the original tool were performed independently by translators with experience in similar tasks. The translators who performed the forward translations synthesized their translations and resolved variations through consensus. We achieved cultural equivalence through the use of an expert committee panel composed of members fluent in both Luganda and English. The Expert Panel Committee comprehensively assessed the translated versions of the tool and the original. By agreeing on words that may not have similar meanings in both contexts or have multiple meanings in our context and ensuring that items inquiring about daily life experiences retain meaning, we are confident that the translated version maintained its meaning in the local context and was also well understood by the participants, as none of the participants in the pilot expressed difficulty in responding to the interview items [24]. Even though there was no quantitative determination of experts’ agreement on the content validity of the translated measurement tool, the qualitative assessment method used is likely to have achieved its purpose, as others have found [25].

The hypothesized three-factor model did not adequately fit or was a poor fit for the data from the 39 items. Subsequently, we performed an EFA [22] with orthogonal varimax rotation of Principal Component Factors and derived a three-factor model that was an adequate fit for the 20 items of the original tool. The resultant shorter tool is important because shorter questionnaires are easier to use for both interviewers and interviewees [22]. Additionally, the shorter version of the measurement tool retained the dimensions/scales from the theoretical framework. For discriminant validity, our results show that the quality of dementia self-care is multidimensional. Covariances between the factors were low, indicating that each factor in the tool measures a distinct but related phenomenon. However, our findings differ from those of Christie et al. [14], who reported a low but negative correlation of PHB with AoC (−0.11, not significant) and PHB with the ECS (−0.38, *p* < 0.001), and a positive correlation of ECS with AoC (0.17, *p* < 0.005) [14, 15]. In our study, PHB was positively correlated with AoC (0.06, *p* < 0.01) and ECS (0.08, *p* = 0.06), with a low but positive correlation between AoC and ECS (0.06, *p* = 0.48). Additionally, we tested the discriminant validity of the quality-of-care tool with the DKAS and found a Pearson’s correlation coefficient of 0.086, indicating that the two instruments measure different constructs.

The adapted tool showed good internal reliability at the factor and full-scale levels. Cronbach’s alpha at the factor/dimension level was 0.77, 0.87, and 0.89 for PHB, ECS, and AoC, respectively, and 0.83 at the full scale. A Cronbach’s alpha of 0.70 and above is acceptable, especially if the instrument is used to measure groups of patients [23]. Christie et al. [14] did not report on the internal reliability of the tool, but a similar study adapting different tools reported similar scores for internal reliability [26].

The findings of this study demonstrate that a shorter, valid, and reliable tool to measure the quality of dementia self-care has been culturally adapted into Luganda and can be used to assess the quality of dementia self-care. Assessing the quality of dementia self-care is important to ensure that people with dementia live well, ultimately improving their quality of life and that of their families and communities. Measuring the quality of dementia self-care is crucial as it helps to identify caregivers providing less than optimum quality of self-care so that they can be supported to improve, and it enhances the prevention of untoward harmful events/effects on care recipients.

Among other possible limitations of this study is social desirability, where participants are more likely to positively respond to statements that reflect high quality of care and negatively respond to statements that reflect poor quality of care. To mitigate against this, we carefully arranged the items in the tool to ensure that we elicit maximum accuracy and response from all participants. We considered PHB items to be sensitive, thus mixed them with others and placed them toward the end of the tool [23].

## 5. Conclusion

The three-factor Quality of Dementia Self-Care Measurement Tool was translated and adapted into Luganda. The results presented show that a shorter, valid, and reliable measurement tool, capable of assessing at least three dimensions of the quality of dementia self-care, has been developed for the local context. The tool can be used by researchers, health workers, and agencies to assess the quality of dementia self-care for older people with dementia. We recommend the use of the measurement tool among the most common languages in Uganda to strengthen its validity.

## Figures and Tables

**Table 1 •:** Study participants’ characteristics

Characteristics	Description	*n* (%)
Sex	Female	66 (62.9)
	Male	39 (37.1)
Age	Median (IQR)	35 (26–47)
Relationship with care recipient	Grandparent	46 (43.8)
	Parent	36 (34.3)
	Spouse	9 (8.6)
	Other	8 (7.6)
	Mother-in-law	5 (4.8)
	Father-in-law	1 (1)
Period caring for care recipient	6 months–1 year	23 (21.9)
	1–2 years	28 (26.7)
	3–4 years	20 (19.1)
	5+ years	34 (32.4)
Attended formal education	Yes	98 (93.3)
	No	7 (6.7)
Highest level of school attended	Primary	44 (44.9)
	Secondary (O level)	35 (35.7)
	Secondary (A level)	14 (14.3)
	Post-secondary— diploma	3 (3.0)
	Post-secondary— degree	2 (2.0)
Tribe	Muganda	75 (71.4)
	Munyankore	13 (12.4)
	Other	9 (8.6)
	Musoga	6 (5.7)
	Mukiga	2 (1.9)
General health condition	Good	32 (30.5)
	Fair	60 (57.1)
	Not good	13 (12.4)
Any chronic ailment	Yes	46 (43.8)
	No	59 (56.2)
Chronic illness	High blood pressure	9 (19.6)
	Chronic backache	9 (19.6)
	Chronic on-and-off headache	7 (15.2)
	Generalized bone aches	2 (4.4)
	Depression	1 (2.2)
	Diabetes	1 (2.2)
	Other	17 (37)
Seeking care for the chronic ailment	Yes	38 (82.6)
	No	8 (17.4)
Where is care sought for chronic illness	Drug shop	20 (52.6)
	Government health facility	11 (29)
	Private health facility	6 (15.8)
	Pharmacy	1 (2.6)

## Data Availability

Data supporting these findings are available within the article, at https://doi.org/10.20935/MHealthWellB7300, or upon request.
